# Using Clinical Vignettes to Evaluate VTE Protocol Adherence

**DOI:** 10.4021/jocmr766w

**Published:** 2012-03-23

**Authors:** Krista Todoric, Erik Lehman, Michael J. Beck

**Affiliations:** a Lehigh Valley Health System, Penn State University, USA; b Department of Public Health Sciences, Penn State University, USA; c Departments of Internal Medicine, Penn State University, USA; d Departments of Pediatrics, Penn State University, USA

## Abstract

**Background:**

Venous thromboembolism (VTE) prophylaxis is underutilized in hospitalized medical patients. Underutilization might occur as a result of resident practice variation incurred by using a complex risk assessment tool.

**Objective:**

To examine what impact repetitive exposure to an electronic point-based VTE risk assessment tool has on resident inter-rater reliability and protocol adherence.

**Design:**

Pre and post intervention cross-sectional cohort study.

**Setting:**

Single academic center.

**Patients:**

Convenience samples of Internal Medicine residents.

**Interventions:**

Residents completed clinical vignettes before and after any exposure to an electronic risk assessment tool and reminder alert. They were asked to make three determinations using a point-based VTE risk assessment tool: risk stratification, identify contraindications, and VTE prevention strategy.

**Measurements:**

Inter-rater reliability for risk assessment, contraindications, and VTE prophylaxis strategy and protocol adherence.

**Results:**

Kappa scores for VTE risk assessment did not change, but improved for VTE plan increasing from 0.28 to 0.37. Protocol adherence improved from 71% in 2008 to 79% (P = 0.06). There was a significant decrease in under-prophylaxis (22% to 6%, P < 0.0001) but a significant increase in over-prophylaxis (7% to 16%, P = 0.001).

**Conclusions:**

Using clinical vignettes, we determined that daily exposure to an electronic risk assessment tool did not improve the inter-rater reliability of a point-based risk assessment tool when used by medical residents. This might be due to inexperienced providers using a complex point-based tool. Overall, adherence improved, and under-prophylaxis decreased, but over-prophylaxis increased. Clinical vignettes are a generalizable method to monitor resident prophylaxis practices and way to identify educational and process improvement opportunities.

**Keywords:**

Resident; Inter-rater reliability; Venous thromboembolism; Agreement; Risk assessment score

## Introduction

Hospital-acquired venous thromboembolism (HA-VTE) is a potentially life-threatening and preventable event that remains a major cause of morbidity and mortality. Despite guidelines being regularly published since 1986 by the American College of Chest Physicians, VTE prophylaxis remains underutilized [1, 2]. As a result, an estimated 900,000 events occur annually in hospitalized patients [3-6]. Current estimates suggest that only 30 - 60% of “at-risk” medical patients receive appropriate VTE prophylaxis [7, 8].

At many medical centers, clinicians assign VTE risk at the time of hospital admission using a point-based VTE risk assessment tool. However, residents perform the majority of admission risk assessments and order VTE prophylaxis; despite few studies showing if residents can reliably apply a VTE risk assessment tool [9, 10]. Reliable application of risk assessment tools can reduce practice variation [11]. In a study where five independent reviewers used a simplified risk assessment tool, the inter-rater reliability for VTE risk level and VTE plan were near perfect and was shown to reduce HA-VTE by 40% [11, 12]. There are no studies that have studied the effect that mandatory and daily use of risk assessment tools might have on the reliability of resident risk assessment, choice of VTE prophylaxis and protocol adherence.

Clinical vignettes have been shown to have many advantages when measuring practice patterns. First, they are useful to monitor changes in physician practice variation after organizational policy changes have been implemented [11]. Second, they are a useful way to examine the extent to which physicians follow these policy changes when isolated from other factors (e.g. reminder alerts) [11, 13]. They are an excellent way to measure practice variation among groups (e.g. residents), and offer the advantages of control for case-mix index and eliminate recording bias inherent in chart abstraction [11]. Finally, they offer more rapid acquisition of data and are less expensive when compared to standardized patients [13].

As more hospitals move towards establishing meaningful use of their electronic health record (EHR), they are integrating VTE risk assessment tools as part of clinical decision support (CDS) with increasing frequency. However, leveraging EHR to implement a CDS tool does not necessarily guarantee protocol adherence, especially if the tool is too complex to reliably apply it [4, 14]. On July 1, 2009, our institution implemented a point-based risk assessment tool that is to be used by residents to facilitate accurate risk assessment and risk level appropriate VTE prophylaxis upon admission. The tool became a mandatory field that required a risk level (low, moderate or high) be assigned to all patients along with a prophylaxis strategy. Reminder alerts were displayed if the prophylaxis strategy did not match the recommendations for patients assessed as moderate or high risk.

Early in the preceding academic year, and prior to institutional integration of the tool, we used clinical vignettes to determine the reliability of the risk assessment tool among a sample of Internal Medicine residents. We found only fair to moderate inter-rater reliability of a point-based VTE risk assessment tool for determining risk assessment and prophylaxis (kappa = 0.51 and 0.28, respectively). This suggests that residents are not likely to come up with same risk assessment and prophylaxis plan for the same patient using a point-based tool early in their training. We hypothesized that the reliability of the VTE risk assessment tool may depend on experience with using the tool, and that reliability would therefore improve over time. In this study, we evaluated the reliability of a point-based risk assessment tool in a sample of Internal Medicine residents one year after integrating of the tool into our EHR.

## Methods

### Study design

#### Clinical vignettes to measure practice variation

We previously reported the inter-rater reliability of a point-based VTE risk assessment tool among a sample of Internal Medicine residents, early in the academic year and without formal teaching. In that study, we used written clinical vignettes to evaluate how residents used the risk assessment tool to risk stratify and determine prophylaxis for the patients in vignettes. We used the same methodology in this study to determine whether test reliability improves among Internal Medicine residents who used the same risk assessment tool for all admissions for a full academic year. Approval was obtained from the Pennsylvania State University Institutional Review Board.

#### Construction of the clinical vignettes

Using data from the electronic health records, we identified individuals > 17 years old who had been admitted to general medicine services via our emergency room. In October 2008, a series of 21 patients was randomly selected and de-identified to portray a range of real-world admission scenarios to be used as clinical vignettes. The vignettes were adjudicated by author MJB. In 2010, to avoid any possibility of recall bias, 15 different vignettes were constructed one year after the electronic intervention was implemented. Thirteen independent reviewers who provided hospital-based care completed the same 15 vignettes. These adjudicated responses were used to determine accuracy of resident risk assessment and appropriateness of residents’ VTE plan.

#### Administration and scoring of the clinical vignettes

We conducted three voluntary one hour long sessions, occurring in October 2008 and May and June 2010 using two different convenience samples of residents from the Internal Medicine program to complete the vignettes. We administered the same 21 vignettes to 23 resident participants (15 interns, 8 senior residents) to determine resident agreement and adherence before the mandatory risk assessment tool was integrated into the EHR. After one full academic year of using the risk assessment tool with an electronic reminder, we administered 15 new vignettes to a new population of 36 residents (14 interns, 22 senior residents) over two one-hour sessions.

All sessions were proctored by author MJB. Time feasibility testing determined that the median time to complete each vignette was 2 minutes 15 seconds (range 30 seconds-7 minutes) per vignette. This was felt to be reflective of the amount of time a resident would spend on risk stratification and prophylaxis planning during an admission and enough time to complete the vignettes during the one hour sessions. At the beginning of each session, the residents received verbal and written instructions regarding application of the VTE tool. They were instructed to make three determinations: risk stratification of each patient, identification of any contraindication(s) to pharmacoprophylaxis, and provision of a VTE prophylaxis plan based on the two prior determinations. VTE plans included 1) ambulation; 2) sequential compression devices (SCDs); 3) heparin product; 4) both SCDs and heparin product, and, in the second session only; 5) continue warfarin. Residents were asked to document any contraindication to pharmacoprophyalxis.

Protocol adherence was determined using the following rules. (Continuation of warfarin was an available option in only 2010).

1. Low risk: appropriate plans were ambulation or continue warfarin if the patient was on it for other medical reasons. Heparin or SCDs was considered over-prophylaxis.

**Figure 1:**
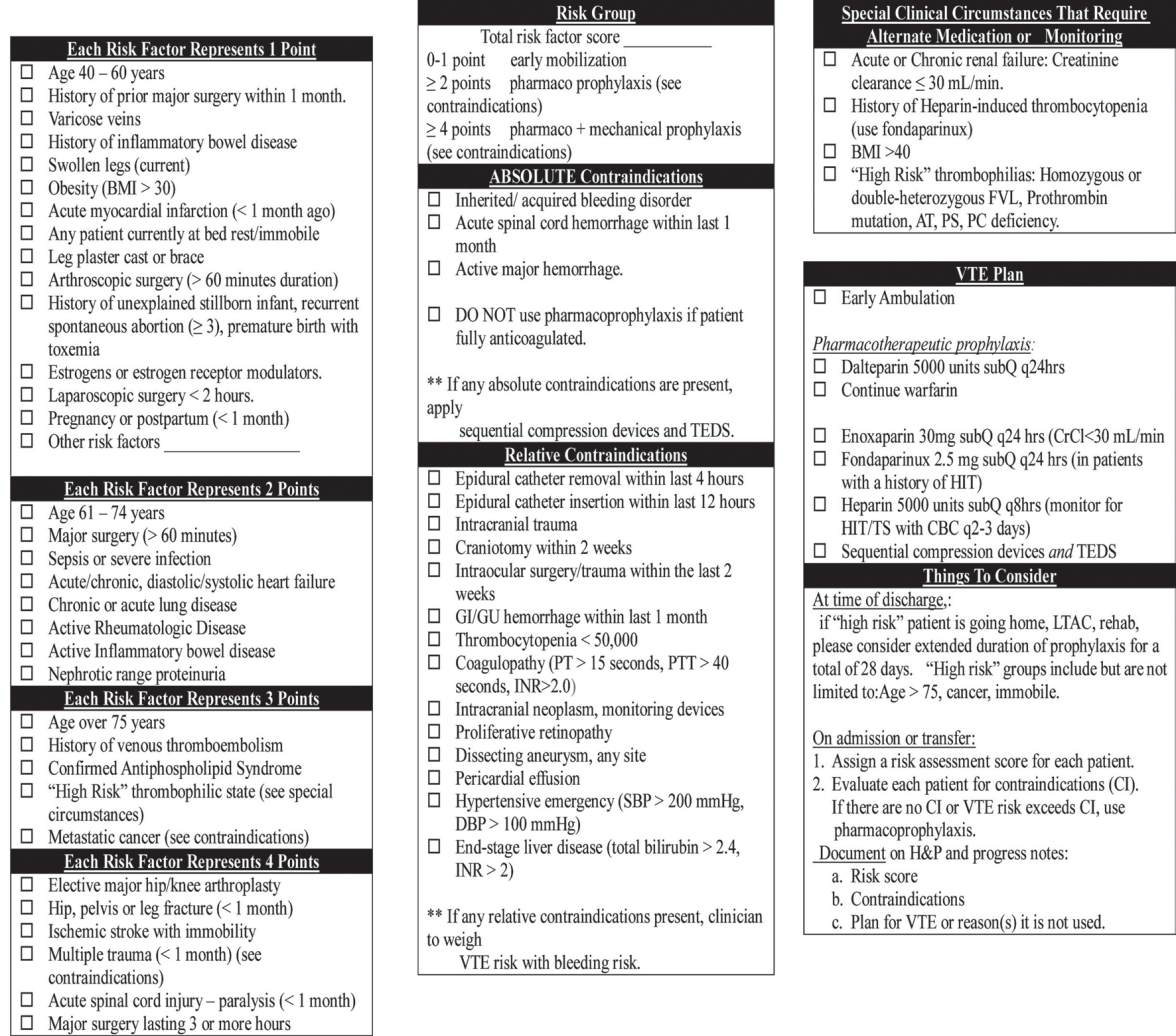
Point-based VTE protocol.

2a. Moderate-risk without contraindication(s): heparin-based therapy was appropriate with or withut ambulation orders (Fig. 1). SCDs ordered in the absence of any contraindication, this was considered under-prophylaxis. Heparin combined with SCDs was considered over-prophylaxis.

2b. Moderate-risk with at least one contraindication: SCDs were appropriate. It was considered appropriate if the patient received pharmacoprophylaxis therapy or was continued on warfarin, because the protocol allows the clinician to weigh VTE risks against bleeding risks (Fig. 1). SCDs and heparin were considered over-prophylaxis. Combining SCDs with ambulation orders was considered inappropriate.

3a. High-risk without contraindication(s): heparin-based therapy combined with SCDs or continuation of warfarin was considered to be appropriate. If there were no contraindication(s), SCDs alone were considered under-prophylaxis.

3b. High-risk with contraindication(s): SCDs were considered appropriate. It was considered appropriate if the patient received pharmacoprophylaxis therapy or was continued on warfarin, because the protocol allows the clinician to weigh VTE risks against bleeding risks (Fig. 1). Combining SCDs with ambulation orders was considered inappropriate.

### Data analysis

We constructed a database using the three variables collected from each resident’s VTE risk assessment form: 1) risk classification (low, medium, or high); 2) the presence of at least one contraindication to pharmacoprophylaxis; and 3) a VTE prophylaxis plan. Fourteen hospital-based clinicians completed the same 15 vignettes in 2010. In power calculations performed prior to the sessions, we determined that the sessions would need at least 300 observations in order to calculate inter-rater reliability (kappa score) [15].

Cohen’s kappa was calculated to assess variability in resident ratings for the following: risk stratification, presence of contraindications, and VTE prophylaxis plan. These ratings were treated as categorical variables. The kappa score has been used in other studies to determine inter-rater reliability using similar VTE risk assessment tools [16, 17]. SAS 9.1.3 was used for all statistical analyses (SAS Institute Inc., Cary, NC).

## Results

### Population

In 2008, 23 residents participated and in 2010, 36 residents participated. Although this represented 33% and 50%, of the internal medicine residents at our institution, respectively, participant response rate was 100%. In the pre-intervention study, a maximum of 483 observations (21 vignettes and 23 residents) was possible. Out of a possible 483 paired assessments and plans, complete data existed for 469 (95%) observations. In the post-intervention study, a maximum of 540 observations (15 vignettes and 36 residents) was possible. Out of a possible 540 paired assessments and plans, complete data existed for 524 (97%) of the observations.

### Risk stratification

In 2008, the residents assessed 72% of patients to be “at-risk” (moderate or high) compared to 85% of a single adjudicator. At the end of the 2010 academic year, the residents deemed 79% of patients to be “at-risk” compared to 82% of adjudicated responses.

To determine the accuracy of resident risk stratification, we compared their risk assessments to adjudicated decisions. There was a significant overall improvement in resident accuracy in risk stratification, improving from 65% in 2008 to 78% in 2010 (P-value 0.015). Comparing the two cohorts for accuracy of risk stratification by risk category, there was no change for low-risk patients (87% in 2008 and 85% in 2010, P = 0.74). However, there was significant improvement noted for both moderate-risk (57% in 2008 and 74% in 2010, P < 0.0001) and high risk patients (59% in 2008 and 78% in 2010, P < 0.0001) (Table 1).

**Table 1:** Comparison of Appropriate Resident Risk Assessments From 2008 to 2010

Appropriate Risk Assessment	2008	2010	P-value
	N = 469	N = 524	
Low	87%	85%	0.74
Moderate	57%	74%	< 0.0001
High	59%	78%	< 0.0001
Overall	65%	78%	0.015

### Risk level appropriate prophylaxis

We used documented contraindication (s) to determine the appropriateness of residents’ clinical judgment when considering patient risk level and their choice of prophylaxis. Comparing 2008 to 2010, overall appropriate VTE prophylaxis occurred in 71% and 79% (P = 0.06) (Table 2), respectively. When we evaluated prophylaxis strategy by risk level, there was a significant improvement in appropriate prophylaxis prescribed to high risk patients in 2010 compared to 2008 (92% versus 69%, P < 0.001). However, fewer low-risk patients received appropriate prophylaxis in 2010 compared to 2008 (68% versus 85%, P = 0.036) (Table 3). There was no change between the two study years for moderate-risk patients (Table 3). Overall, we found that resident under-prophylaxis decreased significantly, from 22% to 6% (P < 0.0001), and over-prophylaxis increased significantly from 7% to 16% (P = 0.001).

**Table 2:** Comparison of Appropriate Prophylaxis Planning From 2008 to 2010

Prophylaxis Plan	2008	2010	P-value
	N = 469	N = 524	
Under-prophylaxis	22%	6%	< 0.0001
Appropriate	71%	79%	0.06
Over-prophylaxis	7%	16%	0.0013

**Table 3:** Comparison of Guideline Adherence From 2008 to 2010

Risk Category	2008	2010	P-value
	N = 469	N = 524	
Low Risk	85%	68%	0.036
Moderate Risk	62%	70%	0.197
High Risk	69%	92%	< 0.001

### Risk stratification agreement

Finally, in regards to inter-rater reliability, aggregate resident kappa score for risk stratification was 0.51 in 2008 (Table 4). In that initial study, we found a trend towards better agreement with increased level of training. The kappa score for interns was 0.47 and was 0.61 for senior residents. In 2010, the aggregate resident kappa score for risk stratification was also 0.51. As in the previous study, agreement improved with increased level of training. The kappa scores for risk stratification for interns and senior residents were 0.45 and 0.54, respectively.

**Table 4:** Comparison of Resident Kappa Scores From 2008 to 2010

	Year	Overall	Interns (R1)	R2	R3/R4
Risk	2008	0.51	0.47	0.61	
	2010	0.51	0.45	0.50	0.54
VTE plan	2008	0.28	0.23	0.35	
	2010	0.37	0.33	0.36	0.39
CI	2008	0.50	NA	NA	
	2010	0.58	0.54	0.57	0.65

### VTE plan agreement

In 2008, the aggregate resident kappa score for VTE plan was 0.28. The same trend was observed for improved agreement with increased level of training. Kappa scores for interns and seniors were 0.23 and 0.35, respectively. In 2010, aggregate resident kappa score improved to 0.37. The kappa scores for VTE prophylaxis for interns and senior residents were 0.33 and 0.39, respectively (Table 4).

## Discussion

Literature suggests that vignettes are useful to track elements of care, especially after changes in process have been implemented [11, 13, 18]. There is also literature that showed repetitive exposure to a computer alert can improve physician performance [19]. Therefore, we performed this study to investigate what impact mandatory and repetitive use of an electronic VTE risk assessment tool might have on resident inter-rater reliability and protocol adherence. We compared previously published inter-rater reliability and adherence data to new inter-rater reliability and adherence data within the same residency program. We found that early in the academic year, and without any exposure to the risk assessment tool, our residents showed variation in risk assessment, VTE plan, and protocol adherence [9]. One year after integrating a point-based prophylaxis tool, the inter-rater reliability remained largely unchanged, but that overall adherence improved. Ours is the first study to show that even with repetitive use of a VTE risk assessment tool aggregate resident inter-rater reliability is moderate for risk stratification, and contraindications and only fair for VTE plan. Aggregate resident adherence to the protocol improved significantly from 71% to 79% (P = 0.06). After one year, residents provided significantly less under-prophylaxis, but significantly more over-prophylaxis. These results corroborate the findings of another study that showed that hospitals that have high prophylaxis rates in “at-risk” (moderate or high risk) patients also have higher over-prophylaxis rates in low risk patients [20]. Finally, data from our 2010 cohort identified that inappropriate use of SCDs with ambulation occurred in 18% of resident plans. This suggests that patients are either receiving SCDs without appropriate documentation of a contraindication, or that hospitalized patients are immobilized because of their injudicious use. This observation identifies specific areas to educate residents, nurses and patients and emphasize the importance of ambulation and to avoid immobilizing patients with SCDs. Since it appears residents learn to better indentify “at-risk” patients through the course of an academic year, but have difficulty appropriately ordering SCDs, educational goals should adjust through the year to meet these knowledge gaps.

As more hospitals work towards improving VTE prophylaxis, some are likely using a point-based risk assessment tool. Many of these same centers rely on residents to use the tool assuming they can use it reliably. This may or may not be true. Prior studies established that a computer alert alone can improve clinician guideline adherence but that there was better adherence when an electronic alert is combined with iterative education [1, 4, 8, 10-12, 19]. Ours is the first study to use clinical vignettes to determine the effect repetitive exposure to an electronic VTE protocol has on resident protocol adherence and the inter-rater reliability of a point-based VTE protocol. Our study suggests that resident accuracy of patient risk stratification and protocol adherence both improve following implementation of a mandatory electronic risk assessment tool, but that inter-rater reliability does not. It also demonstrated that even though there is substantial agreement for contraindication(s), residents still have difficulty using SCDs appropriately in “at-risk” patients.

Our study also confirmed several things we already knew about clinical vignettes. First, they can account for various judgments in a process like: 1) accuracy of risk stratification and prophylaxis plan; 2) protocol adherence; and 3) inter-rater agreement [11, 18]. Second, they identify low performers [11]. This can lead to the creation of learner-focused curricula designed to overcome specific physician-related barriers (knowledge, awareness, attitudes, etc) and improve performance among this group. Clinical vignettes are useful to identify barriers, some of which are knowledge-related, and can be addressed with education. Therefore, like other vignette studies, we suggest efforts be made to intermittently and longitudinally track resident performance [18].

Limitations of this study include that it is a cross-sectional study of two different years of convenience samples of residents that lacks a control group. Therefore, it is possible that the observed improvement in resident performance might reflect trends that routinely occur during the course of every academic year, and might not be attributable to repetitive use of the VTE risk assessment tool. It would have been ideal to compare two randomized cohorts of residents complete the same vignettes at the beginning of the year and at the end of the year with one cohort experiencing the effect of repetitive exposure to the electronic protocol and the second cohort could have served as the control. However, there were several reasons having a control group was not feasible. First, since the VTE tool was integrated into our institution’s EHR as part of a mandatory field to be completed by all residents on all admissions, finding a cohort that would not have been exposed to the VTE risk assessment tool was not possible. A second limitation is that our 2010 cohort included more senior residents than interns. Since residents are better performers than interns it is possible that our results showing improved protocol adherence might reflect the inclusion of a higher performing resident population. However, our 2010 cohort needed to recruit more residents because of the decreased number of observations that would occur as a result of completing six fewer vignettes compared to the 2008 cohort. Thirdly, our study did not compare resident vignette performance to actual resident performance by means of chart abstraction. We chose not to include data abstracted from our EHR since this population of residents would have used a point-based risk assessment tool with the assistance of an electronic reminder alert. This intervention is obviously not available when completing paper-based vignettes and therefore would not reflect resident performance when applying this tool in the absence of an electronic reminder. Finally, as is inherent in any clinical vignette study, it is impossible to overcome the sentinel effect (Hawthorne effect) in which physicians know they are being evaluated [11]. Therefore, the improved resident performance we observed might be an overestimate because they knew their performance was being measured.

Future studies are needed to monitor what effect simplifying the risk assessment process has resident inter-rater reliability for risk stratification and VTE prophylaxis using a control population. A simplified tool that stratifies patients dichotomously as “low” or at-risk “ without a point assignment, might allow residents to spend less time performing risk stratification and more time thoughtfully identifying and weighing contraindications to prophylaxis and use available prophylaxis strategies more appropriately. Ideally, these studies should longitudinally track resident performance every 6 to 12 months. This needs to be balanced with burdening already busy residents with the time it takes to thoughtfully complete clinical vignettes [18]. Our study suggests that excessive heparin exposure and injudicious use of SCDS across all risk categories will occur, and therefore offer the following recommendations. First, resident education should be provided to specifically reduce unnecessary and potentially harmful behaviors. Education highlighting appropriate VTE prophylaxis should occur iteratively throughout the year, starting at intern orientation. Second, residency programs should consider administering vignettes to the residents that will be asked to use a new prediction or decision rule prior to integrating it electronically. For instance, our study identified that the competing orders of SCDs and ambulation and over-prophylaxis of low-risk patients occurs frequently. This will allow information technologists to incorporate electronic rules that will prevent these and potentially other commonly committed resident errors that were only identified by having residents complete vignettes.

In conclusion, as more academic centers implement risk assessment tools, it is important to monitor the effect these interventions have on resident behaviors. This is especially true for academic medical centers, since they are charged with teaching residents standards of practice while simultaneously trying to meet high-stakes institutional quality goals. Whether the risk assessment tool is a part of a paper-based order set or part of a more sophisticated electronic order set, clinical vignettes are a useful, generalizable, and probably under-utilized means in monitoring resident behaviors and can quickly identify unanticipated areas for process and educational improvement.
